# Role of Extracellular DNA in Bacterial Response to SOS-Inducing Drugs

**DOI:** 10.3390/antibiotics12040649

**Published:** 2023-03-24

**Authors:** John K. Crane, Marissa N. Catanzaro

**Affiliations:** 1 Division of Infectious Diseases, Department of Medicine, Jacobs School of Medicine and Biomedical Sciences, University at Buffalo, Buffalo, NY 14214, USA; 2 Jacobs School of Medicine and Biomedical Sciences, University at Buffalo, Buffalo, NY 14214, USA

**Keywords:** RecA, extracellular DNA, antibiotic resistance, polyamines, bleomycin, mitomycin C, aztreonam, antibiotic stewardship, *Escherichia coli*, *Enterobacter cloacae*

## Abstract

The SOS response is a conserved stress response pathway that is triggered by DNA damage in the bacterial cell. Activation of this pathway can, in turn, cause the rapid appearance of new mutations, sometimes called hypermutation. We compared the ability of various SOS-inducing drugs to trigger the expression of RecA, cause hypermutation, and produce elongation of bacteria. During this study, we discovered that these SOS phenotypes were accompanied by the release of large amounts of DNA into the extracellular medium. The release of DNA was accompanied by a form of bacterial aggregation in which the bacteria became tightly enmeshed in DNA. We hypothesize that DNA release triggered by SOS-inducing drugs could promote the horizontal transfer of antibiotic resistance genes by transformation or by conjugation.

## 1. Introduction

The SOS response in bacteria is a conserved stress response pathway that is usually triggered by DNA damage, often due to antibiotics. We and others have noted that non-antibiotic drugs can also be powerful activators of the SOS response, with important implications for antibiotic stewardship. Recombinase A (RecA) is the main sensor of DNA damage and initiates the SOS response by binding to single-stranded DNA, triggering cleavage of the LexA repressor, and thereby stimulating the expression of many genes. The SOS response includes induction of DNA repair pathways, including mismatch repair but also includes induction of alternate DNA polymerases, such as DNA polymerases IV and V, encoded by *dinB* and *umuDC*. These latter polymerases are capable of replicating through stretches of damaged DNA, but they are also error-prone, resulting in the appearance of new mutations. This SOS-associated increase in mutation rate has been called the mutator phenotype, mutator phenomenon, and hypermutation. The new mutations that occur can include those encoding antibiotic resistance [1]. In addition to hypermutation, the SOS response is also accompanied by marked elongation of bacterial cells, which is also termed filamentation [2,3].

Classical inducers of the SOS response include ultraviolet (UV) light and antibiotics such as quinolones and mitomycin C. Quinolones, including ciprofloxacin, inhibit DNA gyrase and topoisomerase, and cause double-stranded DNA breaks, which are strong stimulators of the SOS response. Mitomycin C is a DNA-intercalating dye and also a strong trigger of the SOS response. In addition to the classical inducers, recent work has shown that the SOS response is also triggered by non-antibiotic drugs such as antiviral drugs, anti-cancer drugs, and commonly prescribed antidepressant drugs such as fluoxetine [4,5,6,7,8]. Beta-lactam antibiotics can also induce the SOS response, although disagreement remains about the mechanism [9,10]. Herbicides such as paraquat are also SOS inducers [11]. The use of these drugs in animal and crop agriculture, human medicine, and the environment may be inadvertently promoting the crisis of antibiotic resistance.

In the course of our studies on non-traditional SOS inducers, we compared some of the bacterial phenotypes observed in response to inducers such as the anti-retroviral drug zidovudine, the anti-cancer drug bleomycin, and others. In bacterial cells, as in cancerous tumors, bleomycin causes double-stranded breaks in DNA and activates the SOS response [12]. We compared various inducers for their ability to induce expression of RecA in a *recA-lacZ E. coli* reporter strain, as well as their ability to trigger the emergence of resistance to chloramphenicol and bacterial elongation. We included in our studies a strain of *Enterobacter cloacae*. Although Enterobacter has not been studied as much as *E. coli*, it has interesting properties that make it worthy of greater research attention. These include its well-known inducible, chromosomally encoded ß-lactamase [13,14]. More recently, Enterobacter strains were discovered that had acquired mono-resistance to the carbapenem antibiotic ertapenem [15] while lacking the traditional carbapenemase gene. Recent reports have also shown, alarmingly, that *E. cloacae* can develop resistance to “last resort” antibiotics such as ceftazidime-avibactam and cefiderocol [16,17].

## 2. Results

**Figure 1:**
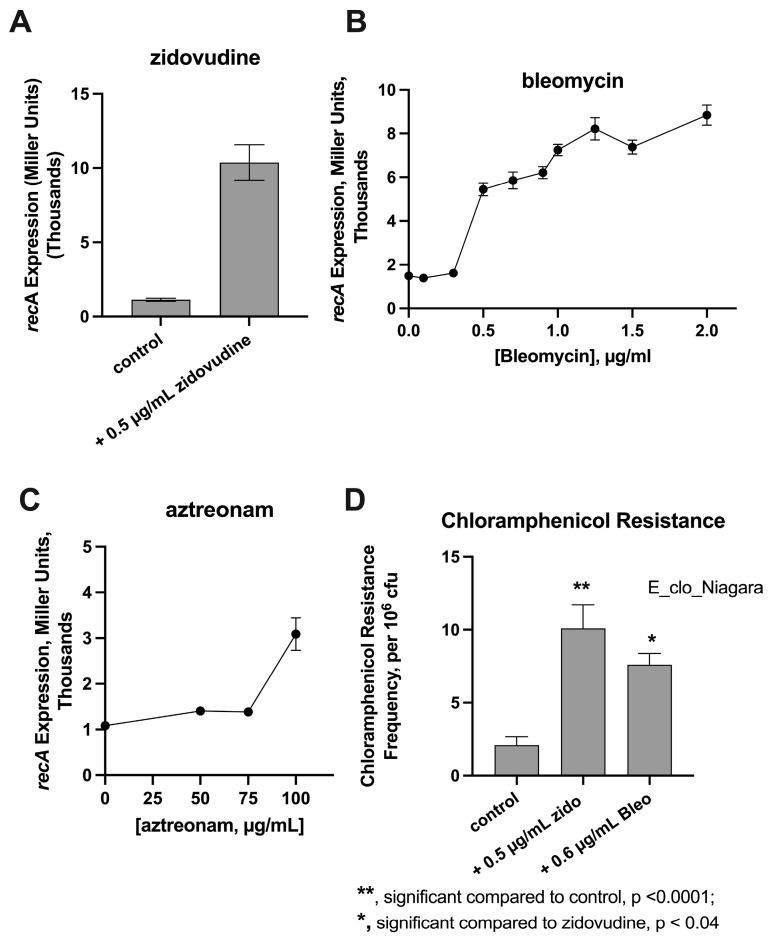
Effect of SOS-Inducers on RecA Activation and Hypermutation. Panels (**A**–**C**), RecA activation as measured using *E.coli recA-lacZ* reporter strain JLM281. RecA activation was measured using the Miller Assay as described in the Materials and Methods. Panel (**D**), Hypermutation assay using zidovudine (zido) and bleomycin (Bleo) as the SOS inducers and using *Enterobacter cloacae* strain E_clo_Niagara. Chloramphenicol, the antibiotic to which resistance was being tested, was added at 22 µg/mL, or 5.5 times the chloramphenicol MIC for this strain.

We began by comparing the ability of various SOS inducers to trigger the expression of RecA using our *E. coli* reporter strain, JLM281 [18]. We previously used this strain to study induction of the SOS response by mitomycin C, 5-Fluorouracil, 5-azacytidine, zidovudine, and ciprofloxacin [11]. As shown in Figure 1A, 0.5 µg/mL of zidovudine, the optimal concentration for this assay, was a strong inducer of RecA. Following a report by Liu et al., we also wished to see if we could confirm that bleomycin caused DNA damage in bacteria and affected the SOS response [12]. Figure 1B shows that bleomycin also triggered RecA expression in the reporter strain. Activation of RecA reached a plateau at about 1 µg/mL of this anti-cancer drug. In contrast, aztreonam was a much weaker inducer of RecA (Figure 1C), even when concentrations of this ß-lactam antibiotic were tested at concentrations well above the MIC. With most drugs, optimal expression of RecA and the SOS response is triggered at concentrations 1/3 to ½ of the MIC, rather than at concentrations above the MIC [19]. We also tested SOS-inducing drugs using a hypermutation assay [11], with chloramphenicol as the drug to which resistance emerged. Figure 1D shows that while zidovudine and bleomycin both increased the chloramphenicol resistance frequency, zidovudine was a stronger trigger of de novo chloramphenicol resistance than bleomycin.

**Figure 2:**
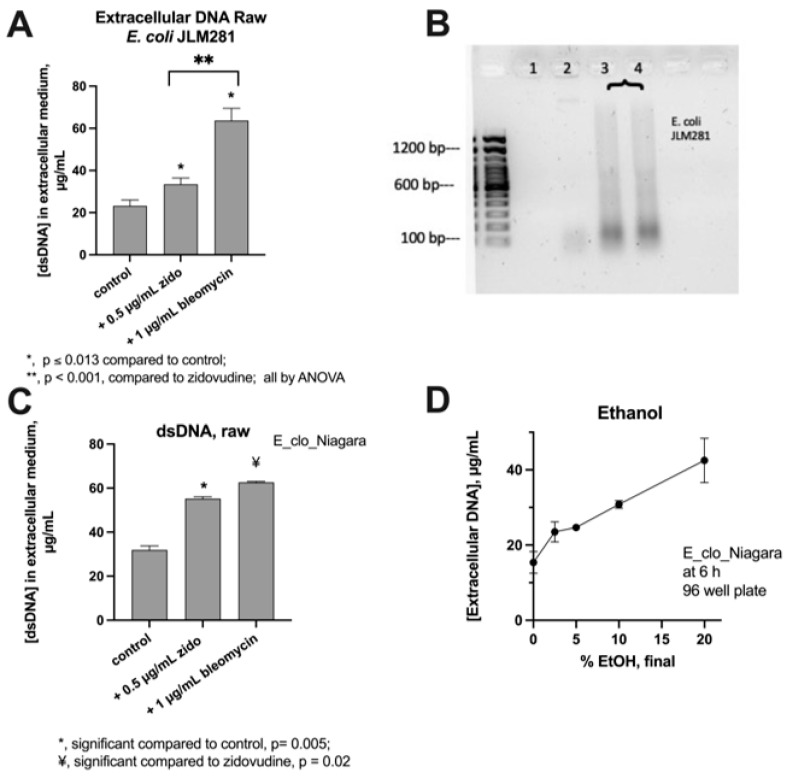
Release if DNA into extracellular media by SOS Induction in *E. coli* JLM281 and *Enterobacter* strain E_clo_Niagara. Panel (**A**), DNA release triggered by zidovudine and bleomycin in JLM281. Panel (**B**), agarose gel electrophoresis of extracellular DNA released from JLM281. Lane 1, untreated control; Lane 2, JLM281 treated with 0.5 µg/mL zidovudine; Lanes 3 and 4, bacteria treated with 1 µg/mL bleomycin. Left lane, 100 bp DNA ladder. Panel (**C**), DNA release from E_clo_Niagara. Panel (**D**), DNA release in response to ethanol treatment. Sublethal concentrations of zidovudine and bleomycin triggered a greater release of DNA than did 20% ethanol, a lethal concentration.

Figure 2 shows experiments to determine if DNA was released into the extracellular medium after treatment of bacteria with SOS inducers. Figure 2A shows that treatment of *E. coli* strain JLM281 with zidovudine and bleomycin both triggered the release of DNA into the medium, but bleomycin-induced DNA release was greater.

Figure 2B shows a DNA agarose gel of the extracellular DNA released from JLM281. DNA released in response to both zidovudine and bleomycin was generally of low molecular weight. As shown in Figure 2B, Lane 2, DNA released from *E. coli* after the zidovudine treatment was 200 bp or smaller in size. DNA released after the bleomycin treatment (lanes 3 and 4) showed a broad range in DNA sizes on the gel, resulting in a “smear,” but with the DNA mostly 1000 bp or less in size.

Figure 2, Panel C, shows the DNA release from the *E. cloacae* strain E_clo_Niagara. For the Enterobacter strain, bleomycin again triggered a greater release of DNA than did zidovudine, and this difference persisted whether the DNA concentration was presented as raw concentrations (Panel C) or adjusted for growth effects by normalizing to culture turbidity (normalized data not shown). For comparison, the DNA-releasing effects of ethanol were tested (Figure 2D), and the ethanol treatment of E_clo_Niagara also triggered DNA release. The amount of extracellular DNA released by 20% ethanol, a lethal concentration, was less than sublethal concentrations of zidovudine and bleomycin (Figure 2A,C).

**Figure 3:**
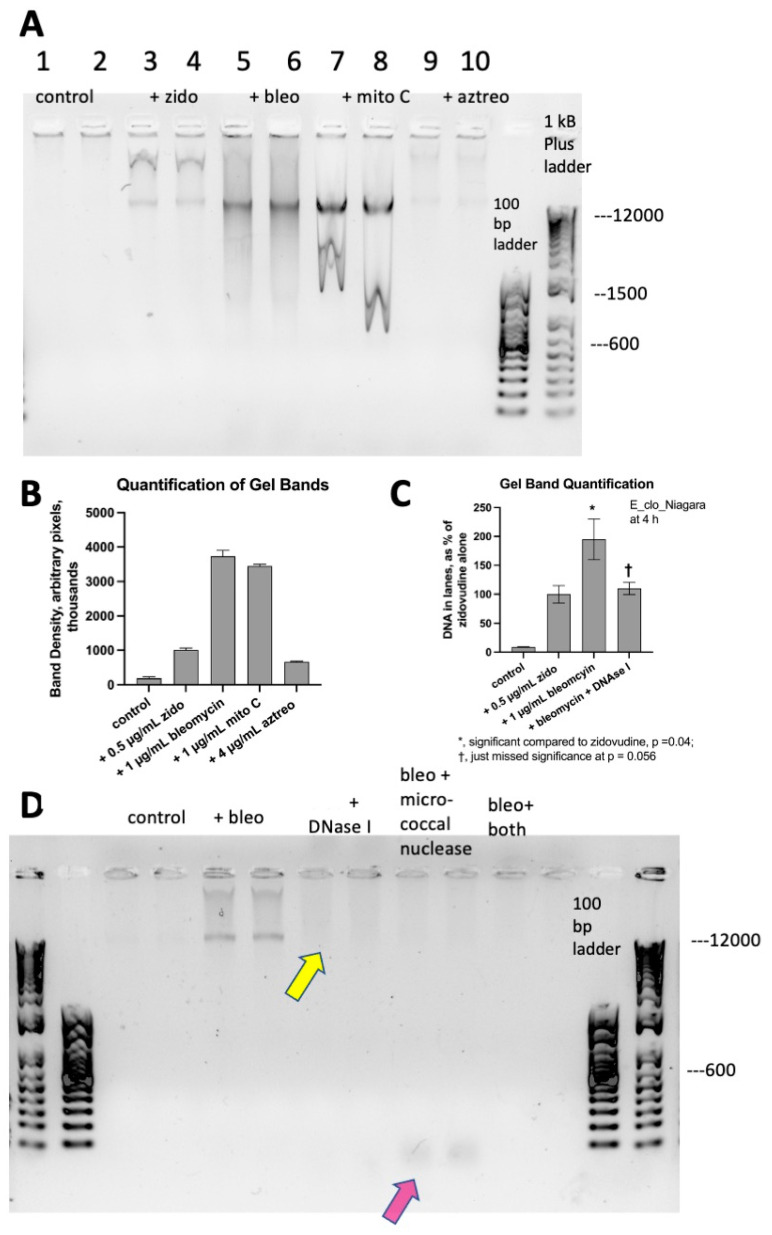
Legend. Panel (**A**), Agarose DNA gel showing differences in DNA banding patterns in response to different SOS inducers, performed in pairs. Panel (**B**), quantification of DNA in lanes from Panel (**A**). Panel (**C**), the effect of DNase I on DNA released in response to bleomycin. Panel (**D**), Agarose DNA gel showing the effect of DNase I and micrococcal nuclease on DNA release after the bleomycin treatment, again in pairs. DNase I treatment left some larger DNA fragments in the sample (yellow arrow). Micrococcal nuclease digested most of the DNA, but with small fragments seen (pink arrow). The combination of DNase I and micrococcal nuclease eliminated DNA from the samples (“bleo + both”).

Figure 3 shows additional experiments investigating DNA release from E_clo_Niagara. Figure 3A shows the differences in the amount of DNA released as well as the differences in banding patterns of DNA released in response to different SOS-inducing drugs. Zidovudine triggered the release of larger DNA fragments (Figure 3A, lanes 3 and 4). Mitomycin reproducibly triggered DNA with the unusual “W-shaped” bands seen in lanes 7 and 8. Figure 3B shows the quantification of DNA in the lanes from Figure 3A. Figure 3C shows the effect of adding DNase I to the E_clo_Niagara cultures. DNase I treatment reduced but did not completely eliminate the DNA observed in the supernatant medium (Figure 3C). Therefore, we tested if the addition of a different nuclease, micrococcal nuclease, would have a different or additive effect. Micrococcal nuclease acts on RNA as well as DNA and is an exonuclease as well as an endonuclease. In contrast, DNase I only has endonuclease activity and only acts on DNA. Figure 3D shows that DNase I reduced the amount of bleomycin-induced DNA in the supernatants, with some DNA remaining (yellow arrow). The addition of micrococcal nuclease alone abolished the larger DNA fragments, but small DNA fragments, ~100 bp, remained (pink arrow). The addition of both DNase I and micrococcal nuclease eliminated DNA in the samples. The differences in the DNA banding patterns after treatment with DNase I and micrococcal nuclease suggested that the DNA released by SOS inducers may vary in its susceptibility to different nucleases, perhaps due to differences in conformation.

**Figure 4:**
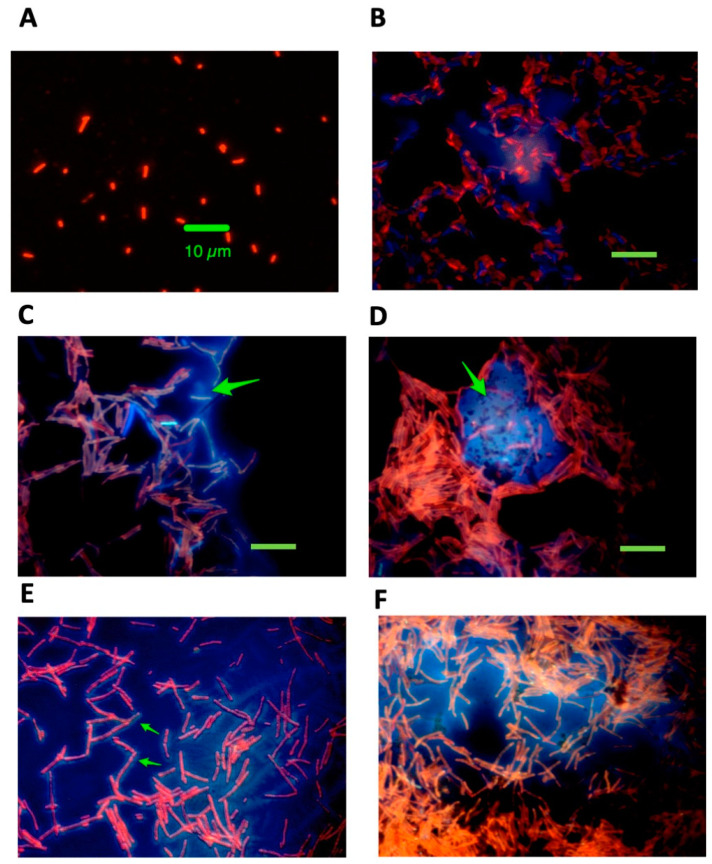
Legend. Dual staining of E_clo_Niagara with and without exposure to zidovudine, exogenous DNA, and DNase I. Bacteria were double stained with acridine orange and DAPI as described in the Materials and Methods and examined using fluorescence microscopy at 1000× magnification. Panel (**A**), untreated control. The green size bar indicates 10 µm, and this size marker applies to all the panels in Figure 3. Panel (**B**), E_clo_Niagara treated with 75 µg/mL DNA alone and with faint traces of blue-fluorescing DNA visible. Panel (**C**), bacteria treated with 0.5 µg/mL zidovudine alone, showing bacterial elongation, bacterial clumping, and the appearance of blue-fluorescing amorphous extracellular material closely attached to bacterial cells. The green arrow indicates the presumed extracellular DNA. Panel (**D**), bacteria treated with zidovudine + 50 µg/mL exogenous DNA, showing the appearance of massive clumps of bacteria tightly wrapped around the blue, DAPI-labeled extracellular material (green arrow). Panel (**E**), same condition as Panel D but in an area of the slide where bacteria were less dense. In Panel (**E**), the extracellular DNA appears to be coating the elongated E_clo_Niagara bacterial cells (green arrows). Panel (**F**), E_clo_Niagara treated with zidovudine, exogenous DNA, and DNase I. Bacterial cells were still elongated, and bacterial clumps were still present, but the clumps appeared less dense than in Figure 3D.

We next used double labeling of the Enterobacter strain with Acridine Orange and 4′,6-diamidino-2-phenylindole (DAPI) to visualize whether the release of DNA into the extracellular medium had effects on bacterial morphology. Figure 4 shows fluorescence microscope photographs, all at 1000× magnification.

Figure 4A shows the control, untreated Enterobacter, strain E_clo_Niagara, where the bacteria are about 2 µm in length, on average. Figure 4B shows E_clo_Niagara treated with 75 µg/mL of DNA, and only a faint trace of DAPI staining material was still present after the two washings, and no bacterial elongation was seen. Figure 4C shows the appearance of the bacteria after treatment with 0.5 µg/mL zidovudine. Bacterial elongation, a well-known part of the SOS response, was clearly visible. In addition, diffuse DAPI-staining extracellular material could be visualized clinging to the bacteria (blue color, green arrow), and the bacteria showed more aggregation than in Panels A and B. Figure 4D shows a typical field in bacteria treated with zidovudine plus DNA. Massive clumps of E_clo_Niagara were present, and they often surrounded areas of DAPI-staining DNA (green arrow). In areas on the slides where the bacteria were less dense, the DNA appeared to surround the bacteria rather than the other way around (Figure 4E), where the arrows indicate elongated bacteria apparently coated with a thick layer of DNA.

When bacteria were treated with zidovudine, DNA, and DNase I (Figure 4F), the clumps of bacteria appeared less dense, but DAPI-staining material was still present. Although this persistence of DNA was unexpected, it is consistent with what we observed in Figure 3, Panels C and D, in which treatment with DNase I reduced but did not fully eliminate the extracellular DNA.

**Figure 5:**
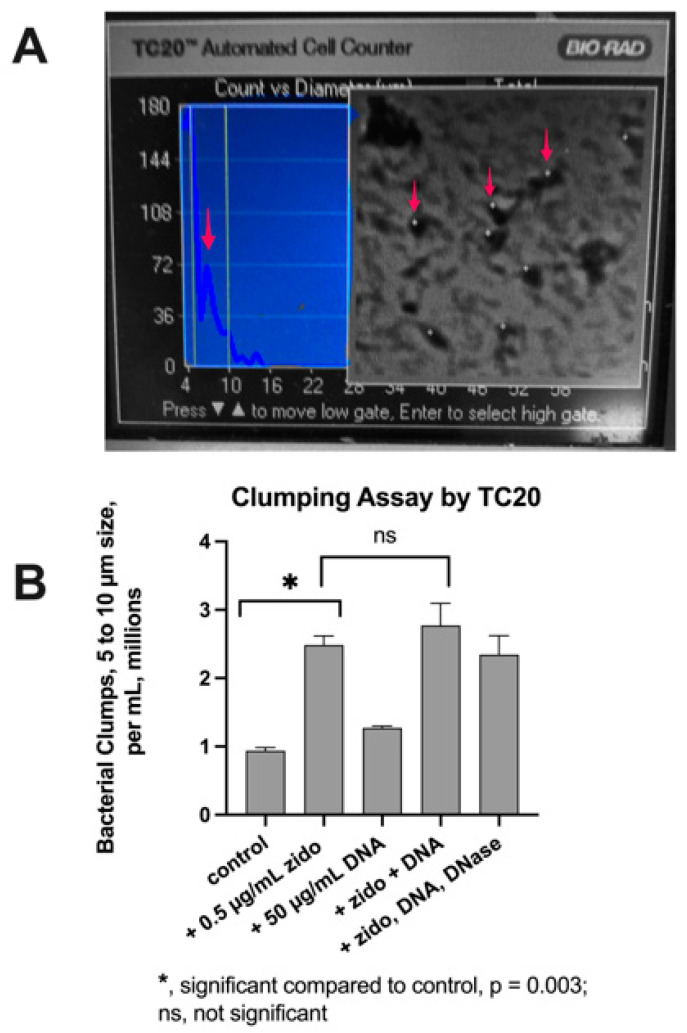
Quantitative Analysis of Bacterial Clumping. Panel (**A**), photograph of the TC20 screen showing the detection of clumps between 4 and 10 µm in size. Red arrow at left shows a peak of detected objects. On the right, red arrows indicate objects detected by the TC20counter, which are marked by the instrument with a small white + sign. Panel (**B**), results of the counts of an experiment with E_clo_Nagara. DNase I was added to a final concentration of 20 U/mL.

We sought to quantitate the clumping phenomenon that we observed in the E_clo_Niagara bacteria in Figure 4. We used a cell counter intended for counting mammalian cells to detect clumps of bacteria significantly greater than the Enterobacter cells themselves. Figure 5 shows that we were able to adapt the TC20 cell counter to detect bacterial clumps. Figure 5A shows a peak in bacterial aggregates detected with the counter (left side of Figure, red arrow). The right side of Figure 5A shows the appearance of the clumps detected with the instrument; the clumps are marked with a small white dot (actually a small “plus” sign, +). The quantitative results of this analysis are shown in Figure 5B. The addition of 0.5 µg/mL zidovudine significantly increased the number of bacterial clumps observed per mL. Adding exogenous DNA alone did not have any significant effect on clumping.

Zidovudine + DNA seemed to show a slight increase in clumps compared to zidovudine alone, but this failed to reach statistical significance. The addition of DNase I had a modest effect (right-hand column of Panel B), but DNase failed to abolish the clumping below the level observed with zidovudine alone.

**Figure 6:**
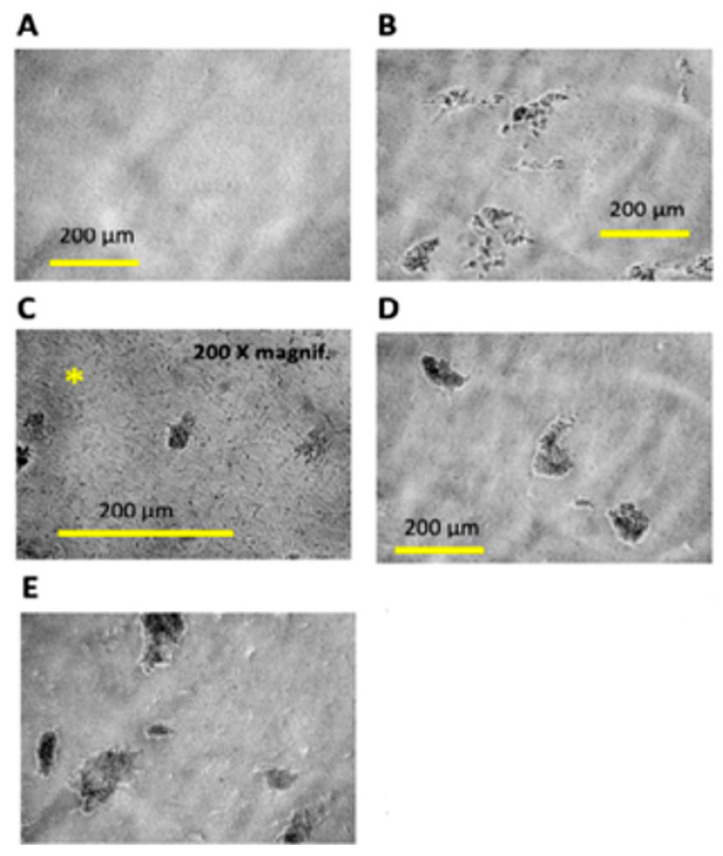
Detection of SOS-Induced Clumping by Phase Contrast Microscopy, in E_clo_Niagara. Panels (**A**,**B**,**D**,**E**) were photographed at 100 × magnification, while Panel (**C**), was at 200 × magnification. Panel (**A**), control; Yellow Size Bar indicates 200 μm; Panels (**B**,**C**), treated with 0.5 μg/mL zidovudine; Panel (**C**), yellow asterisk highlights bacterial elongation. Panel (**D**), 0.5 μg/mL zidovudine + 50 μg/mL DNA; Panel (**E**), treated with 0.5 μg/mL zidovudine + 20U/mL DNase I.

After conducting the experiments with the TC20 cell counter, we suspected that the size of the bacterial clumps was large enough that they could be detected using microscopy under low power without fluorescent dye staining. Figure 6 shows photographs of E_clo_Niagara taken at 100× magnification using Phase Contrast microscopy. Figure 6A shows that untreated, control bacteria were barely visible at this low power. When E_clo_Niagara was treated with zidovudine alone, the formation of loose bacterial clumps was observed (Figure 6B). At higher 200× magnification, bacterial elongation was visible (asterisk) along with the bacterial clumps (Figure 6C). When the Enterobacter were treated with zidovudine + exogenous DNA, the clumps appeared denser and more refractile, and many of the clumps exceeded 30 µm in size (Figure 6D). Treatment with zidovudine + DNase I again failed to reverse the clumping phenomenon (Figure 6E). We next tested if SOS-inducing drugs triggered a release of cytoplasmic contents. We tested this in two ways. First, we labeled E_clo_Niagara with Calcein AM, the acetoxymethyl ester of calcein. When the ester moiety is hydrolyzed by intracellular esterases, the active fluorescent compound is released. Supplemental Figure S2 shows that the calcein dye was retained intracellularly in response to SOS inducers such as zidovudine (Figure S2, Panel B).

In contrast, calcein seemed to leak from the cells in response to aztreonam (Figure S2, Panel C). We also used an *E. coli* strain, strain EC43, which expresses green fluorescent protein (GFP). In EC43, GFP was retained intracellularly in response to both zidovudine and aztreonam (Figure S2, Panels D and E). Our experiments with the calcein AM dye and with GFP showed that SOS-inducing drugs did not cause a bulk release of cytoplasmic contents into the extracellular medium.

## 3. Discussion

While investigating other aspects of the SOS response, namely the possible role of polyamines in SOS induction [20], we discovered that substantial amounts of DNA are released into the extracellular medium by SOS inducers. The literature on the SOS response is voluminous, but there has been scant attention to SOS-associated DNA release, although there have been some hints that DNA release might occur [12,21]. We found that DNA release from bacteria was triggered with exposure to many classic inducers of the SOS response, including zidovudine, mitomycin C, bleomycin, and ciprofloxacin. The extracellular DNA may have several biological activities, but one such activity appeared to be bacterial aggregation or clumping. DNA alone was unable to trigger bacterial clumping, but it appeared to increase the size of the bacterial clumps in cells also exposed to zidovudine (Figure 4D and Figure 6D).

One reason for continued strong interest in the SOS response is its role in promoting antibiotic resistance [6,7,22]. Release of DNA by an antibiotic-resistant bacterial strain might be one way that such resistance could be transferred to a different, more susceptible strain, as in bacterial transformation in naturally competent bacteria [23,24]. The size of the DNA fragments released in our experiments, however, was small (Figure 2B and Figure 3A,D). The small fragments that we observed, mostly of 1500 bp or less, are not big enough to encode an entire antibiotic resistance gene. However, these fragments, despite their small size, seem capable of promoting close bacteria-to-bacteria adherence. By bringing bacterial cells into close contact, DNA could promote the horizontal transfer of resistance genes by a different mechanism, namely conjugation. Activation of the SOS response promotes a conjugative horizontal gene transfer [25,26], although the possible role of extracellular DNA was not noted in those reports. In our study on SOS-induced antibiotic resistance in vivo in the rabbit intestine, we found that non-pathogenic “bystander” bacterial strains could become enmeshed in the extracellular DNA triggered by infection with an enteropathogenic *E. coli* strain, strain E22 [22]. In those experiments, we assumed that the extracellular DNA was all of host cell origin, in the form of neutrophil extracellular traps, or NETs [27,28,29,30]. Our current work indicates that, in an infection, DNA of bacterial origin could be co-mingled with host DNA. This could occur even in the absence of antibiotic treatment, as oxidant host defenses are strong inducers of the bacterial SOS response [31,32,33,34].

One notable feature of our findings was that DNase I did not have as dramatic an effect on DNA measurements and on bacterial clumping as one might have expected. Addition of DNase I did appear to reduce the amount of DNA present in the extracellular medium (Figure 3D). One possible explanation is that the extracellular DNA released by the bacteria becomes enmeshed tightly in bacterial aggregates in a manner that renders the DNA less accessible to the DNase enzyme, which is an endonuclease. The addition of micrococcal nuclease, which is active on RNA as well as DNA, and which has exonuclease and endonuclease activity, seemed to act additively with DNase I to fully digest DNA in the bacterial suspensions.

Liu et al. reported that DNA-induced bacterial aggregation required DNA in the 100 to 500 µg/mL range and a 24 h incubation [35]. In contrast, in our experiments with bacteria exposed to SOS-inducing drugs, ~ 50 µg/mL of DNA was sufficient (Figure 6D), and large bacterial clumps appeared just 2 h after the addition of the SOS inducers (see the Materials and Methods). Induction of the SOS response changes the lipids exposed on the surface of *E. coli* bacteria [36] and causes membrane depolarization [37,38] both of which could make the bacteria more “sticky” toward negatively charged extracellular DNA. Gram-negative bacteria usually have a net negative charge due to the negatively charged phosphates in lipopolysaccharide (LPS). The SOS-triggered alterations in surface lipids, however, could reduce repulsive electrostatic forces and allow interaction between DNA and bacterial cells.

Many recent articles have noted that extracellular DNA plays an important role in the formation of biofilms, including in pathogenic bacteria [30,39,40,41]. A smaller number of articles have shown that the SOS response also promotes the formation of bacterial biofilms [42,43], but those reports did not recognize that the two phenomena are linked. Our results reported here, therefore, provide a bridge between these two areas of investigation because the SOS-inducing drugs actually trigger the release of extracellular DNA.

A second finding of our study was the marked differences between different drugs in inducing various aspects of the SOS response. As shown in Table 1, there appeared to be a good correlation between the ability to induce RecA activation and the ability to induce hypermutation in vitro in the Enterobacter strain. In contrast, there was no correlation, or at least no positive correlation, between the ability of drugs to induce DNA release and to induce the bacterial elongation response (Table 1, columns 4 and 5). In fact, there may even be an inverse correlation between DNA release and bacterial elongation, since aztreonam, which triggered massive bacterial elongation (Supplemental Figure S1, Panels D, F, G, and H) was the drug showing the weakest DNA release (Figure 3B). More inducing drugs will need to be tested, however, to determine if this possible inverse relationship between DNA release and elongation is a general phenomenon.

## 4. Materials and Methods

### 4.1. Bacterial Strains and Growth

The bacterial strains used are shown in Table 2. In general, strains being tested for SOS induction were grown overnight in LB broth at 37 °C with 300 rpm shaking, then subcultured 1:100 into DMEM/F12 medium, which was chosen because it enhances expression of the SOS response. Bacteria were subcultured in the DMEM for 1.5 h, also at 37 °C with 300 rpm shaking, before the addition of SOS inducers because rapidly growing bacteria are more susceptible to this stress response. At the 1.5 h point, exogenous DNA, and enzymes such as DNase I, were also sometimes added. Bacterial suspensions were then collected at 3.5 to 4 h for analyses such as RecA expression, DNA release, clumping, staining with fluorescent dyes, and so on.

### 4.2. Reagents Used

Acridine orange, chloramphenicol, 4′,6-diamidino-2-phenylindole (DAPI), DNA from salmon testes, HEPES buffer, LB EZ Mix agar, and zidovudine were from Sigma-Aldrich (now Millipore Sigma). DNase I, from the bovine pancreas, and micrococcal nuclease, were also both from Sigma-Aldrich. Aztreonam and bleomycin were from Cayman Chem, Ann Arbor, MI. Agarose for DNA gels was SeaKem LE agarose from Lonza Co., Rockland, ME. Antibiotic MIC Strips were from Liofilchem, Waltham, MA.

DMEM/F12 powder was from the Gibco Division of Thermo-Fisher, Grand Island, NY, USA. The composition of this medium is available at https://www.thermofisher.com/us/en/home/technical-resources/media-formulation.329.html, accessed on 16 March 2023. The DMEM/F-12 was supplemented with 18 mM NaHCO_3_ and 25 mM of additional HEPES, pH 7.4. Sybr Safe DNA stain, 100 bp DNA ladders, and 10 X loading dye (“Blue Juice”) were from the Invitrogen Division of Thermo-Fisher.

### 4.3. Miller Assay for RecA Expression

We used our *E. coli* reporter strain, JLM281, to measure the induction of RecA as previously described [5,45]. This assay uses the production of ß-galactosidase, under the control of the *recA* promoter, and is adapted to a 96-well plate format [46]. Please note that in this assay, bacteria do not have to remain viable at the end of the assay as long as the ß-galactosidase enzyme has been produced.

### 4.4. Hypermutation Assay

We used the methods reported previously used to determine if drugs would induce the hypermutation response [11,22,26]. Strain E_clo_Niagara is resistant to rifampin, however, so we instead used chloramphenicol as the drug to which resistance was being induced. We used chloramphenicol at 22 µg/mL, which is 5.5 times the MIC for this Enterobacter strain.

### 4.5. Electrophoresis of DNA

DNA Electrophoresis on agarose gels was as previously described [26,45], in which 1 to 1.5% agarose was used in 1 X Tris-acetate- EDTA buffer (1X TAE). We added equal volumes of bacterial supernatants to the wells in each gel experiment, usually 25 µL of the sample mixed with 10 X loading dye. Fluorescent DNA bands on gels were visualized using SybrSafe dye and photographed using the Gel Doc EZ instrument (Bio-Rad, Hercules, CA, USA) and the Image 5 software. Quantitation of gel bands and gel lanes was performed using the Un-Scan-It Gel program for the MacIntosh by Silk Scientific (Orem, UT, USA).

### 4.6. Measurement of DNA NanoDrop UV Spectrophotometer

DNA released from bacteria was measured using the settings for double-stranded DNA (dsDNA) on the NanoDrop instrument (Thermo-Fisher). In some samples, DNA measurements were verified using the Qubit Fluorometer (Thermo-Fisher), and the fluorescence assay confirmed the NanoDrop results. DNA was analyzed directly from the culture supernatants and was not concentrated or purified using kits or columns.

### 4.7. Staining of Bacteria for Fluorescence Microscopy

Bacteria were stained using 0.025% acridine orange and 12.5 µg/mL diamidino-phenyl indole (DAPI). Acridine orange stains DNA, RNA, and protein, while DAPI is specific for DNA. Rapidly growing bacteria usually stain orange with Acridine Orange [47,48]. Fluorescently stained bacteria and DNA were examined at 1000× magnification. In some cases, an image obtained using the Acridine Orange filter was superimposed on the image obtained using the DAPI filter to form a merged image. The program HDRtist NXL, version 2.0.4, was used to superimpose two images (Ohanaware Co., Taipei, Taiwan).

### 4.8. Visualization of Bacterial Clumps with Low-Power Microscopy Using Phase Contrast

Bacterial clumps could be visualized without staining using Phase contrast microscopy at 100× or 200× magnification.

### 4.9. Quantitation of Clumping Using the TC20 Cell Counter

We used the TC20 Cell Counter (Bio-Rad), intended to measure mammalian cells, to measure the appearance of bacterial clumps greater than 4 µm in size. The TC20 instrument uses microscope slides and image analysis software to detect objects.

### 4.10. Data Analysis

Graphs were created using GraphPad Prism, version 9.5.0, and this program was also used for statistical calculations. A *p*-value of ≤0.05 was chosen as is usual in biomedical research.

### 4.11. Ethical Approvals

No humans or animals were used for this research, so no approvals were needed.

## 5. Conclusions

The SOS response is a potent inducer of new antibiotic resistance. However, the SOS response has been studied mostly by research microbiologists interested in the fascinating mechanisms involved. In contrast, the SOS response has not been emphasized by those working in fields such as clinical microbiology, clinical infectious diseases, antimicrobial pharmacy, and antibiotic stewardship. However, as additional research findings accumulate, the era in which the SOS pathway can be safely ignored by clinicians may be swiftly passing away.

## Figures and Tables

**Table 1:** Differences Between Various SOS-Inducing Drugs in their SOS Phenotypes. Combining results with *E. coli* JLM281, *E. coli* Popeye-1, and *Enterobacter cloacae* E_clo_Niagara.

SOS Inducer	RecA Activation	Hypermutation	Extracellular DNA Release	Elongation	Comments
zidovudine	+++	+++	++	+++	See Figure 4
bleomycin	++	++	+++	++ *	See Supplemental Figures S1 and S2 and Videos
ciprofloxacin	+++	+++	++	+++	Supplemental Figure S1, and previous study [11]
mitomycin C	+++	Not tested	++++	++ *	
aztreonam	+/−	Not tested	+/−	+++++	Supplemental Figure S1

*, with bleomycin and mitomycin C, a minority of the bacterial cells, estimated at less than 25%, showed marked elongation, while many other cells were normal or near normal in length.

**Table 2:** Bacterial Strains Used.

Strains	Description	Comment	Reference(s)
*E. coli* Strains			
JLM281	*recA-lacZ* reporter strain	Used to measure RecA expression using Miller assay; Aztreonam MIC is 0.047 µg/mL on LB.	[11,18]
Popeye-1	Shiga-toxigenic *E. coli*, O157:H7; 2006 spinach-associated outbreak	Stx2+, Stx2c+ TW14359	[34,44]
EC43	Shiga-toxigenic *E. coli*, O157:H7; Fluorescent strain due to GFP	From Microbiologics; traceable to FDA strain ESC1177; used to assess leakage of cytoplasmic contents.	[26]
Enterobacter Strain			
E_clo_Niagara	Wild-type clinical isolate, bloodstream	ESBL; Chloramphenicol MIC = 4 µg/mL, and Aztreonam MIC = 3 µg/mL, both on LB.	[26]

## Data Availability

Not applicable.

## Supplementary Materials

The following supporting information can be downloaded at: https://www.mdpi.com/article/10.3390/antibiotics12040649/s1, Supplemental Figure S1: Bacterial elongation using SOS inducers in Enterobacter and *E. coli*; Supplemental Figure S2: Retention of Intracellular Dyes after treatment with SOS inducers; Video S1: control culture; Video S2: bleomycin-treated culture.
