# Supplementary material for: Role of Extracellular DNA in Bacterial Response to SOS-Inducing Drugs
Source: Antibiotics (Basel). 2023 Mar 24;12(4):649. doi: 10.3390/antibiotics12040649 (PMC10135224; doi:10.3390/antibiotics12040649)
Supplement: Supplementary file 1 [file antibiotics-12-00649-s001.zip › Supplem Figs. S1& S2.pdf]

Supplemental Figure S1

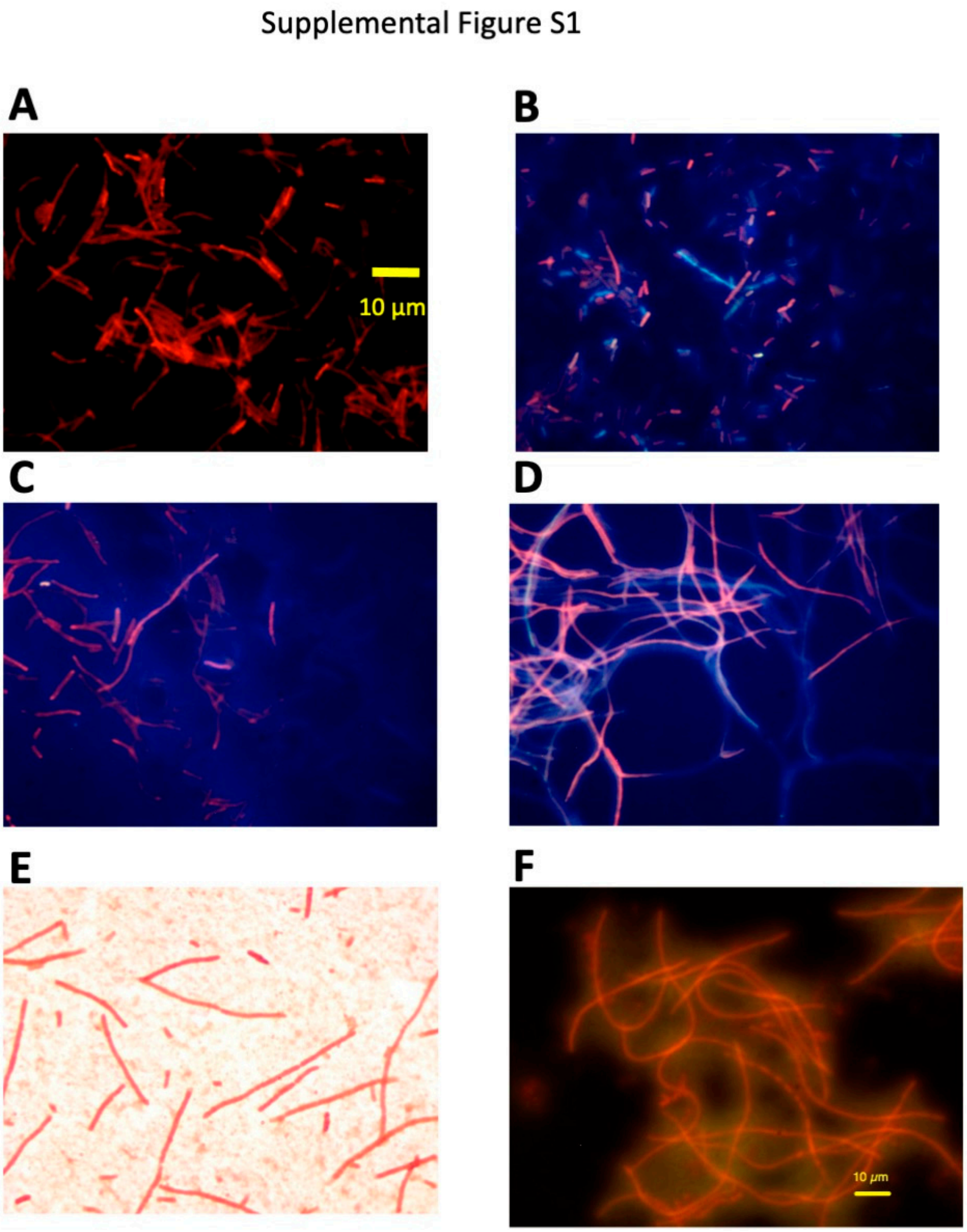

Supplemental Figure S1, continued

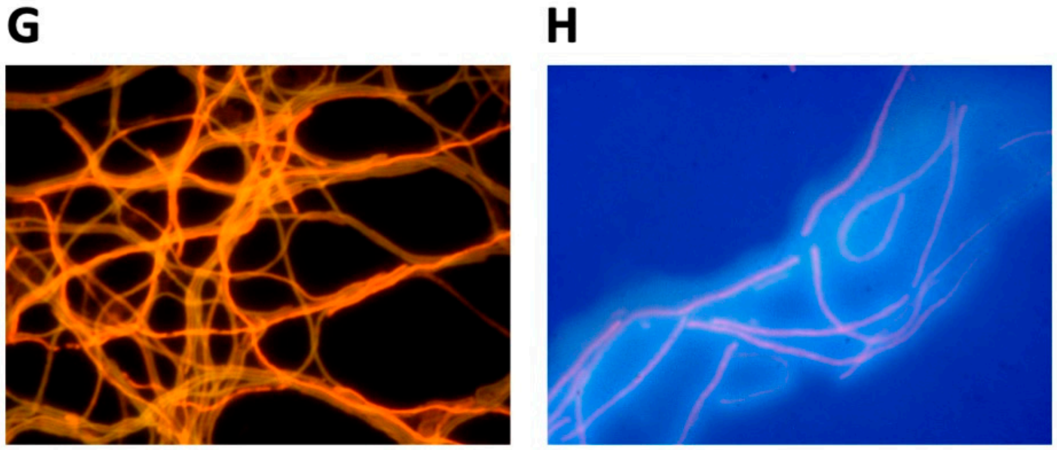

Supplemental Figure S1. Legend.
Elongation Response of Bacteria to Various SOS Inducers.

All Panels were photographed at 1000 X Magnification under Oil.
Panels A- D show photographs of *E. cloacae* E_clo_Niagara, and Panels B-D are double-stained with Acridine Orange and DAPI. Panel A, E_clo_Niagara treated with 40 ng/mL ciprofloxacin, Acridine Orange stain; size bar shows 10 µm, which applies to all panels. Panel B, bacteria treated with 1 µg/mL bleomycin. Panel C, treated with 1 µg/mL mitomycin C; Panel D, treated with 4 µg/mL aztreonam. Panel E, *E.coli* JLM281 treated with 40 ng/mL ciprofloxacin, Gram stain; Panel F, JLM281 + 1 µg/mL aztreonam;
Panel G, Shiga toxigenic *E. coli* (STEC) strain Popeye-1 treated with 4 µg/mL aztreonam, Acridine Orange stain, showing extreme elongation. Panel H, Popeye-1 treated with 4 µg/mL aztreonam plus 50 µg/mL DNA, Double stained with Acridine Orange and DAPI. Aztreonam fails to trigger DNA release, but if exogenous DNA is supplied, it will still become enmeshed around the STEC bacteria.

Supplemental Figure S2

Supplemental Fig. S2

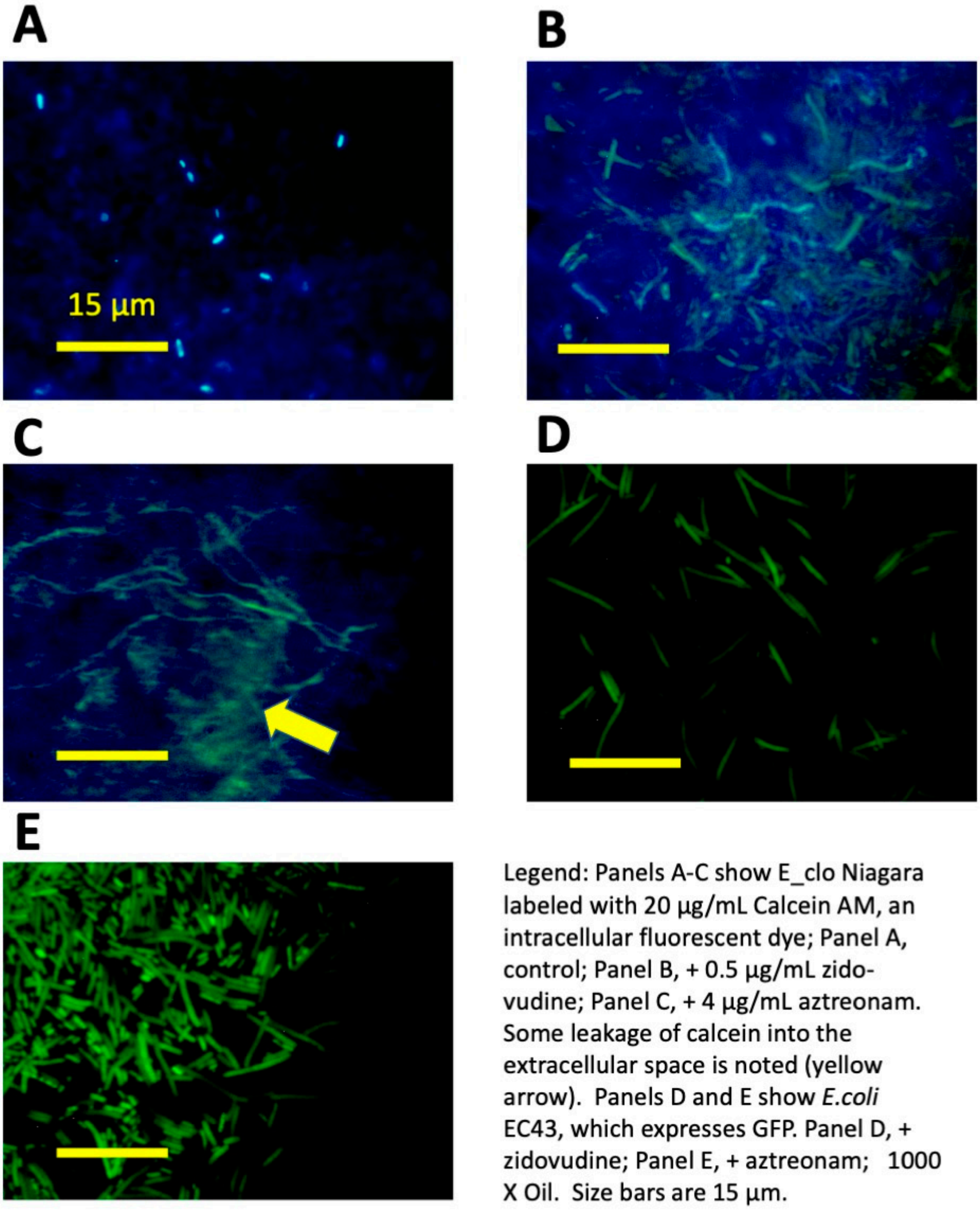

Legend: Panels A-C show E_clo Niagara labeled with 20 µg/mL Calcein AM, an intracellular fluorescent dye; Panel A, control; Panel B, + 0.5 µg/mL zidovudine; Panel C, + 4 µg/mL aztreonam. Some leakage of calcein into the extracellular space is noted (yellow arrow). Panels D and E show *E.coli* EC43, which expresses GFP. Panel D, + zidovudine; Panel E, + aztreonam; 1000 X Oil. Size bars are 15 µm.
